# “It made me feel human”—a phenomenological study of older patients’ experiences of participating in a team meeting

**DOI:** 10.3402/qhw.v8i0.20714

**Published:** 2013-05-28

**Authors:** Elisabeth Lindberg, Ulrica Hörberg, Eva Persson, Margaretha Ekebergh

**Affiliations:** 1 School of Health Sciences, University of Borås, Boras, Sweden; 2 School of Health and Caring Sciences, Linnæus University, Växjö, Sweden; 3 Board of Health Sciences, Faculty of Medicine, Lund University, Lund, Sweden

**Keywords:** older persons, phenomenology, team meeting, patient participation, qualitative research

## Abstract

This study focused on older patients participating in a team meeting (TM) in a hospital ward in Sweden. A process had taken place on the ward, in which the traditional round had developed into a TM and understanding what participating in a TM means for the older patient is necessary for the development of care that facilitates older patient's participation. The aim of this study was to describe the caring, as experienced by the older patients on a ward for older persons, with a specific focus on the team meeting. A reflective lifeworld research (RLR) design was used. Fifteen patients, 12 women and three men (mean age of 82 years) were interviewed while they were hospitalized in a hospital ward for older people. In the essential meaning of the phenomenon, the TM is described as being a part of a wider context of both caring and life. The need for hospitalization is an emotional struggle to overcome vulnerability and regain everyday freedom. The way in which the professionals are able to confirm vulnerability and create a caring relationship affects both the struggle for well-being and the possibilities for maintaining dignity. The essence is further explicated through its constituents; Vulnerability limits life; Life is left in the hands of someone else; Life is a whole and Space for existence. The result raises concern about how the care needs to be adjusted to older people's needs as lived bodies. The encounter between the carer and the patient needs to be developed in order to get away from the view of the patient as object. An expanded vision may open up for existential dimensions of what brings meaning to life. One way, as described by the patients, is via the patient's life stories, through which the patients can be seen as a whole human being.

This study focused on older patients participating in a team meeting (TM) in a hospital ward in Sweden. A process had taken place on the ward in which the traditional round had developed into a TM. Fifteen patients who had experienced a TM were interviewed and observations preceded 12 of the 15 interviews.

A major focus in the scientific literature on the round appears to be communication and interaction between the physicians and patients (Molony, Horgan, & Graham, 2013; Weber, Stöckli, Nübling, & Langewitz, 2007) and interaction in the health care team (Manias & Street, 2001; Weber et al., 2007). The literature also focuses on the ward round as being important for the education of staff and students (Bradfield, 2010; Manias & Street, 2001; Molony et al., 2013; O'Hare, 2008).

Patients often experience the round as fragmented and brief, leaving little time for interaction (Persson & Määttä, 2012; Sweet & Wilson, 2011). Communication during rounds can be challenging due to professionals using medical terms or due to the lack of the opportunity for confidentiality when sharing a room with other patients (Molony et al., 2013). Subjects addressed during the round mainly concern medical issues (Weber et al., 2007).

Changing and developing the ward round is described as a challenging task due to the inherent and hierarchical structures it relies on (Fiddler et al., 2010). Historically, ward rounds have a high status in hospital care and courage, vision and leadership is needed in order to reform the ward round (Bradfield, 2010). Several areas for improvement have, however, been suggested such as: developing the patient perspective (Manias & Street, 2001; Molony et al., 2013; Sweet & Wilson, 2011; Weber et al., 2007), evaluating alternatives to the “bed to bed” round (Fiddler et al., 2010; O'Hare, 2008) and developing a more inter-professional team work during the round (Bradfield, 2010; Fiddler et al., 2010; Manias & Street, 2001; Weber et al., 2007).

TMs have been described as one way of developing the round and different contexts in which TMs occur are, for example, palliative care (Parker Oliver, Porock, Demiris, & Courtney, 2005; Wittenberg-Lyles, Parker Oliver, Demiris, & Courtney, 2007), care of older people (Blesedell Crepeau, 2000; Gair & Hartery, 2001; Jones & Jones, 2011) and psychiatric care (Fiddler et al., 2010; Vuokila-Oikkonen, Janhonen, & Nikkonen, 2002). The aim and frequency of a TM vary relating to its context and the persons involved in the meeting. Involvement of different professions and having the intention to work as a team seems, however, to be of importance (Fiddler et al., 2010; Jones & Jones, 2011) and whether the patient and family members count as team members or not can differ. In some studies, the patients’ involvement in TM is described as important but challenging (Opie, 1998; Parker Oliver et al., 2005; Vuokila-Oikkonen et al., 2002). The TM was initially a place for professionals to discuss the treatment and care of the patients in the ward where this study took place. The question whether to invite the patients to participate was subsequently raised; the aim being to increase the possibilities for patient participation and to strengthen the patient perspective in care and discharge planning.

Research has proven that inviting a patient to a TM demands reflection on how to involve the patient in the situation. Communication, especially concerning emotional subjects, seemed to be difficult for the professionals to cope with (Parker Oliver et al., 2005; Vuokila-Oikkonen et al., 2002). Creating a dialogue with the patient is also described as demanding due to the traditional patterns of communication in which the professionals are seen as experts (Opie, 1998). To our knowledge, there are few studies that take into consideration the experiences of older patients participating in a TM. Reviewing literature about other contexts may contribute to increased understanding for the challenges that can occur in the meeting between the older patient and health care professionals. Efraimsson, Sandman, and Rasmussen (2006) describe how older women taking part in a discharge planning conference (DPC) experienced ambivalent feelings in the situation. On the one hand they felt affiliated and in focus, while on the other they had feelings of not knowing what was expected of them and a feeling of being outside the situation. Rydeman, Törnkvist, Agreus, and Dahlberg (2012) describe how a biomedical perspective, in which the patient's complex situation was not explicit, led to the older persons who took part in the DPC feeling stressed and contributing to a loss of coherence. Older people are often very conscious of the difficulties they have in participating in their care due to illness (Ekdahl, Andersson, & Fredrichsen, 2010). Hearing loss, impaired vision and the need for more time to think and speak can, for example, make appointments with the physician a challenging experience for the older person (Summer-Meranius, 2010). Thus, based on the following challenges: the patient's unclear role at the TM, the staffs’ difficulties in meeting the patient's need from a holistic perspective, challenges connected to the patient's ageing body and the limited amount of research on how older patients experience the TM, there is a need for further research in this field. Experiences of older patients taking part in TM are valuable in order to further develop the care; the phenomenon of this study is thus “caring with specific focus on the TM”.

## Aim

The aim of this study was to describe the caring, as experienced by the older patients on a ward for older persons, with a specific focus on the team meeting.

## Method

The study has a descriptive design and is based on the theoretical framework grounded in lifeworld phenomenology and caring science, which can in this study be seen in the interest for the older patients’ lived experience of the TM. The research process was guided by the phenomenological approach of reflective lifeworld research (RLR) (Dahlberg, Dahlberg, & Nyström, 2008). This is based on the phenomenological philosophy of Husserl (1970/1936; 1977/1929) and Merleau-Ponty (1968/1964; 2011/1945). The major characteristic of RLR is a reflective attitude of openness and adherence to the (meaning of the) phenomenon and an act of “bridling” towards that which appears throughout the research process. The act of bridling can be seen as thoughtfulness throughout the procedure in which the understanding and search for meaning has to be slowed down “by a kind of active passivity” (Dahlberg et al., 2008 p. 122) in order to allow the phenomenon to show itself in its uniqueness. The researchers have strived to maintain an open (i.e., bridling) attitude during the research process with critical questioning among the members of the research team as well as in seminars.

## Participant and setting

The study was conducted in a ward for older people at a hospital in western Sweden. The planned hospitalization on the ward would ideally not exceed three days. The ward was characterized by a holistic patient perspective, where the patients and their life situation were in attention. The TM functioned as the core arena for the planning of the care to be given on the ward and the discharge from hospital. The staff had tried to improve patient participation by inviting patients to the TM. Fifteen participants (12 women and 3 men) were interviewed in this study. All of them lived in an ordinary housing and as a majority had some kind of support from the home care services, planning for discharge was of importance in order to create a safe departure from hospital. All of the interviews were conducted while the informants were hospitalized for an acute somatic illness. The informants were 74–94 years (mean age of 82 years) and three lived together with a wife or a husband.

## Data collection

The patients were invited to participate in the TM by their nurse or physician. When the first author was present on the ward, the patients were also asked by their nurse/physician if they wanted to participate in the subsequent study. Patients who wanted to participate were then contacted by the first author who provided information, both orally and written. This first contact, due to the patient's vulnerability, served as a way of establishing a relationship in which the informants could feel safe and ask questions. Patients with impaired decision-making skills (for example, due to a diagnosis of dementia or patients affected by fatigue or illness) were excluded.

Observations preceded 12 of the 15 interviews. Observations can, according to Dahlberg et al. (2008), be complementary to interviews when the phenomenon “is social and inter-subjective, embedded and implicit, and therefore hard to get hold of, as hard to verbalize” (p. 219). Field notes were taken during the observations; these are not, however, included in the analysis, but served as a basis for reflection during the interviews and can serve as background information and contribute to a better understanding of the result. One example taken from an observation and interview with a woman is described here. During the TM, the patient starts to cry and the nurse assistant sitting next to her puts her arms around her. During the interview, the patient was asked to describe her experience of the situation and she then described her tears as tears of gratitude “… they were so nice to me and then I was sorry for that too ….”

The interviews took place immediately after the TM and followed the principle of RLR (Dahlberg et al., 2008). The two main questions were: How did you experience the TM? and Can you please tell me why you are in need of hospital care? Follow-up questions were then asked in order to gain a greater understanding of the phenomenon: “caring with specific focus on the TM”.

## Analysis

The data were analysed according to the principles of RLR (Dahlberg et al., 2008), which means that reflection and bridling have been vital components throughout the process. The interviews were transcribed verbatim by the first author and non-verbal information such as, for example, silence and hesitations were included, as described by Dahlberg et al. (2008). Listening to and transcribing the interviews contributed in the process to becoming familiar with the material. The analysis started by reading and rereading the interviews to create a feeling for the material as a whole. Thereafter, the text was divided into “meaning units”. The analysis proceeded by focusing on understanding the meaning of every unit. This was carried out through thoughtful reading where single words, sentences or paragraphs related to the phenomenon were identified. Questions related to the phenomenon were asked of the material, such as: how and in which way is caring present in the described TM situation. In the next phase, the meanings were put together in clusters. A cluster is a temporary pattern, supporting the researchers in the search for an essential meaning (Dahlberg et al., 2008). In RLR, the essence is the most abstract level of analysis (Dahlberg, 2006). The essence was formulated by rereading the clusters as well as the original data. A continual movement back and forth between the parts and the whole led to the emergence of the essence and meaning structure. Once the essence had been formulated, the analysis continued by identifying the more contextual nuances of the phenomenon, which in RLR is termed as the constituents.

## Ethical considerations

The study was approved by the Regional Ethical Review Board of Gothenburg (757-09). The study was carried out in accordance with the principles outlined in the Declaration of Helsinki (World Medical Association Declaration of Helsinki, 2008). The informants were informed both orally and in writing (the information letter was on some occasions read to the informant) that participation was voluntary and that confidentiality would be maintained. Written informed consent was obtained from each informant. Consideration of the informant's condition was made, for example, signs of fatigue were taken seriously resulting in the interview being concluded. The researcher also made sure that the informants had understood the meaning of participation in the study. This was mainly carried out by talking with the informants about the aim of the study and by encouraging questions about the information given.

## Results

The results are presented in a structure of meanings, where the essential meanings are initially described and then followed by the constituents of the phenomenon: Vulnerability limits life; Life is left in the hands of someone else; Life is an entity and Space for existence.

The TM is a part of a wider context of both caring and life. When hospitalization occurs “real life” takes a break. Freedom and independence in everyday life is put into brackets when the need to surrender to the hands of other humans and enter into a patient role occurs. When vulnerability increases or becomes overwhelming, relationships with other humans become a driving force providing meaning and support in life. In caring and during the TM, there is a longing and a desire to be recognized and confirmed as a unique, but vulnerable human. The professionals have an important role in how the caring and the TM is experienced by the patient. The way in which the professionals are able to confirm the patients’ vulnerability and create a caring relationship affects both the struggle for well-being and the possibilities for maintaining dignity. The TM is an emotional meeting concerned with life and existence. Joy and gratitude for the opportunity of resuming life is mixed with fear facing an uncertain future and grief over lost opportunities. The need for hospitalization is an emotional struggle to overcome vulnerability and regain everyday freedom. The patient's natural strength is weakened when the vulnerability takes over and the struggle is brought to a standstill. If a positive approach to life is maintained then the struggle to regain everyday life can be strengthened. The motivation is a longing to leave the break from “real life” to regain freedom and to resume life.

A focus on the present and the future means that the past is confined to medical history and the life story remains unseen. Space for the whole human has to be conquered; otherwise life as experienced previously is excluded from the present and not included in the future. Fragmentations of the historicity reduce the holistic approach and prevent the creation of meaning. A long life creates experiences and attending the TM is reflected on in relation to experiences in life and in previous encounters with the health care system.

## Vulnerability limits life

During the TM, the patients describe an increasing vulnerability, in which fatigue and powerlessness emerge and take a great place in their lives. The body restricts the opportunity of leading their everyday lives in the same way as they have done previously. During hospitalization, the longing for an independent life, becomes a driving force in the fight against vulnerability “… a hospital is good and I'm grateful that it exists so that one can get help … but home is always the best, so that if you don't think that home is best, then you must be ill!” Furthermore, the relationship with other people and being able to continue to provide care constitutes motivation in the struggle against vulnerability and contribute to the feeling of having a mission to fulfill. A woman exemplifies this by describing how she had to give up one activity after another and hand over her care to others. What she is still able to manage and what is of great importance is to continue to take care of her cat. In an existence where the ability to perform previously, taken for granted activities, decreases, the cat serves as a proof that she is still needed and has a meaningful task to fulfill.

The patients struggle to sustain life, but everything from severe illness to feelings of exhaustion may cause the patient to step over the limit of their ability to independently manage the situation. One limit is passed when the need for home care service arises. The situation at home and the need for support in daily life is often discussed during the TM. For the professionals, the discussion is mainly a matter of practical solutions, for example, which aids the patient needs. For the patient, there is an existential dimension in which the vulnerability is exposed through the need for home care. The transition from being the one who takes care of, to the one being taken care of is transformative “For me it's hard to realize that I'm so ill that I need help! Because I want to help others.” Not being able to care for oneself and becoming a burden generates feelings of shame. There is a cognitive understanding of the need for support, but feelings and experiences are associated with grief over lost abilities and limited freedom in everyday life. For those patients who already have been in contact with home care service, the experiences vary from a sense of security to something that restricts their freedom, takes energy and causes feelings of insecurity prior to discharge.

The vulnerability present in everyday life is sometimes reinforced in the caring context. Just moving to the room where the TM takes place can entail a great deal of effort. Fatigue, impaired vision and hearing isolate the patient and it can be hard to maintain attention and take an active part in the situation “… it was when the doctor turned to me and spoke to me that I heard, but otherwise I could hear that they were talking, but what it was about I couldn't say ….” This situation exemplifies a patient being present in the room, but her possibility for taking active part in the conversation is limited.

## Life is left in the hands of someone else

A picture emerges in which the patients surrender to the care. An understanding is required of what it means for the patients to surrender, as one purpose of the TM is to actually enable the patients to regain control over their lives. Surrendering means security and rest in some situations, while in others it means giving up one's freedom and sometimes oneself also. When the need for hospital care occurs, life takes a break and the patient role is given priority over the normal everyday one. Taking a break means, for example, that everyday routines are put aside, despite the fact that they can contribute to well-being. A patient describes how he avoids walking in the corridor due to a fear of not being allowed to “… I miss not being allowed to exercise … I try to walk, but I'm a little bit worried that I'm not allowed to do that ….” What is important in everyday life does not disappear but remains as a goal to achieve when the need for care does not exist any longer. Longing for home, the everyday routine and the familiar environment are powerful motivators.

It has sometimes been necessary and vital to surrender when the disease has created a need for other people and their expertise. When the patient has been seriously ill, the health care services have almost completely taken over the patient's life “I remember when the ambulance arrived, but after that I can't remember any more.” A void is created in which the patient has been excluded from other humans. By asking questions and talking to the staff, the patient tries to recreate what has happened and to bring meaning to the void. The need to surrender to other people also occurs when strategies in everyday life are deficient. Ingenuity and strength are needed in order to get control over life with an ageing body and its illnesses. Sometimes the limit is passed when the patient's own ability becomes insufficient and the whole situation becomes overwhelming. “… I have had pain in my back for two years, so now I thought: I can't stand one more day!” The limit, as described by the patients, is not a clearly defined point in life where everything changes rapidly. The pain, as described by the patient in the quotation above, has been endured for a long time but at some point the patient runs out of energy and cannot fight alone any longer. It is when this unobservable limit is passed that the need to surrender to care arises.

In a surrender that is characterized by trust, the patient experiences that the staff acts in their best interests. When a patient feels trust s/he can remain passive and allow the staff to make decisions concerning their care as well as the planning for their discharge. In a trustful surrendering, the patient can rest and gather strength and confidence for the future. Confidence is built through the staff responding to the patients’ need for care. However, a passive surrender may also contain traces of resignation. The patient neither can nor has the strength to make up her mind and instead allows herself to be guided by the staff. A woman describes it as “… they (the staff) know how to treat me” and she continues “it was good to attend (the TM) and hear what they said … but it's not easy to have an opinion ….” The patient is able to mobilize sufficient strength to be present and to listen, but participating in a more active way is difficult and demands more energy.

## Life is an entity

A long life provides rich and diverse experiences. The present is shaped by experiences in all facets of life and remains as memories, stories and emotions. The TM's focus on the present and the future means that the past lived life is overlooked. Sometimes not only the history but also the future dimensions fall away, leaving a one-dimensional focus on the present. When the future dimensions are not included, what has been determined during the TM is not carried out and the patient's trust in the staff is reduced and the patient then questions the meaning of participating in the TM. A patient describes how she prefers having her sleeping pill earlier than the routine time at the hospital. The woman addresses the issue during a TM and it is decided that she is to receive her sleeping pill at a time suitable for her. The decision is never carried out in practice and the woman has to continuing waiting for her sleeping pill “… I'm almost ready to go to sleep … then I took the (sleeping) pill, but it was too late! So I have to read for a while … in order to be tired again.”

The medical anamnesis has its given place during the TM and is given much space, while the meaning of living with the illness and the way this affects life is obscured. The patients, however, describe the importance of telling their stories, in which they can emerge as unique human beings and not just reduced ageing bodies. What is important to share varies; some patients describe that they have lived with the disease most of their lives.

… I have worked my whole life, but I had a really tough childhood, it was during the war and I was born on the west coast and I stood and cleaned cold fish and it was so cold! It was then a lot of my pain … but at twenty-five I discovered that I was not able to move the way I should have been able to.

Life in the shadow of illness affects choices made in life and contributes to shape the type of person you are today. In the TM's focus on the present looking towards the future, the patients speak of the whole picture being neglected and not asked for “… a lot depends on how your childhood was and how your marriage was, and they (the staff) were just asking: How is it at home?”

Historicity also involves emotions in a substantial way. It is in relation to one's own history that the current situation emerges and becomes evident. Limitations in life, caused by ageing and illness, are seen by the patient in relation to the abilities and dreams that s/he had earlier in life and which to a great extent still remain. This comparison with her/his previous life often leaves the patient with emotional grief over the losses; physical ones as well as losses of significant others.

An invitation to the TM can be experienced as an invitation to something unpredictable. Being uncertain as to what is expected of the patient during the TM can create anxiety “I was a little bit worried before … thought I might not be able to answer ….” One way of gaining an understanding of what participation in a TM means, is to reflect on the unknown (the TM) in relation to something known (experiences from earlier encounters in care). Experiences can also be based on participation at institutional conversations such as the DPC. The experience affects how the patient feels towards the TM. Previous encounters characterized by respect can generate a sense of security, while an experience of a disrespectful encounter can lead to feelings of suspicion. A woman describes how she, because of previous experiences, prepares herself to, as she puts it “defend herself” during the TM “… verbally I can defend myself … I had that in mind and thought that they would not do that against me again.” Experiences from previous care episodes are sometimes from many years previously, when the role of the patient was described as more subordinate than today “I had a ruptured appendix when I was 16 years old … you had a great respect for doctors and hospitals and that feeling is still there.” The patients bring emotions from previous encounters into the TM, and the TM situation is compared and assessed with previous encounters used as references.

Participation in the TM is sometimes described as being uncomplicated. Earlier experiences from life contribute to a sense of security and the patient dares to occupy space in the situation. Experiences from a professional life that included meetings create feelings of safety and comfort during the TM. Living alone for different reasons also demands independence “… I've been a widow for 26 years now and of course I've had to make decisions on my own very often.” The opportunity to participate in the TM is a way of maintaining independence even in the patient role.

## Space for existence

The patients have their own beliefs about their situation; beliefs that can cause worry and create a reduced well-being. Concerns about how the future will turn out are predominant in their lives. The patient's ability to be active is limited when anxiety prevails “When you are alone at home you often start to brood about things, and then it's as though you lose it, there's so much that I have done before … then you don't feel like doing it when you are worried …” Anxiety limits not only the ability to be active but can also manifest itself in physical symptoms such as dyspnoea. “Because of the pain I worked myself up when I was at home … I thought I couldn't breathe.” The possibility for the patient to be able to describe their anxiety is important for their experience of well-being. From the patient's perspective, it is important that the professionals show an interest in and create opportunities for the patient to tell her/his story during the TM. One way of doing this might be by posing questions to the patient, which can make it legitimate for the patient to take the space. When the opportunity for the patient to say what is important for her/him is limited or there is a complete absence of questions, the patient is left with the feeling that the professionals are not interested in gaining a greater understanding of their life situation. A patient describes how he perceived the professionals attending the TM as “silent and shy”. The patient had expected more questions from the staff and when there were no questions this was interpreted by the patient as being disinterested “they (the staff) just got up and walked away.”

Loneliness and anxiety are examples of great challenges in life for the patient. One way of taking the edge off the worst fears and creating relief is when the patient is provided with space to be able to describe her/his feelings and to ask questions during the TM. It can also create conditions for dealing with anxiety in everyday life. Many of the questions asked by the patient have a deep existential meaning, for example, questions such as: “Will I be able to carry on with my life?” can be hidden behind questions about test results and drugs. The importance of taking the patients’ narrative seriously emerges in the following way “I was afraid about my legs and you can see that it can be so bad that they may need to amputate … But then she explained to me, that this was not how it was in my case.”

To be present at the TM, listen to the discussion and be the only one in focus entails a confirmation and creates feelings of exclusiveness. A patient describes how participating in the TM made her “feel human” and she continues:

Yes, first it is this that it is about me, it feels good to be involved as well … and even say what you think. I think I'm quite dependent on it … I have done this all my life … both in my job and at home and everything. I have never needed to be outside ….

A positive image of the ward is revealed in the narratives, which is often described in terms of “feeling at home”. The homelike feeling is created by the staff's attitude: “small things” such as looking after the patient and asking how s/he feels is of great value “It was the girls! They came in and comforted me and talked and yes they were amazing! … it was just as if I had been at home!” The patients also describe how important it is not to feel as though they are a burden to the staff as well as to be sensitive towards the latter's signals in terms of verbal as well as non-verbal language. The descriptions of feeling at home also include feelings of safety and confirmation, as well as trust in the fact that the staff are there for them. The homelike feeling also contributes to the feeling of being in a safe place when attending the TM.

## Discussion

The informants had individual needs and expectations related to the care, but ageing and illness had caused a need for hospital care. During their stay in hospital, they found themselves in an alien environment in which they were always a step behind and in the hands of the professionals. Both the care and the planning for discharge and the future are carried out on others’ terms. It has been found that adjusting the care to meet with the patients’ experiences and hopes for the future can result in feelings of them being valued and treated with dignity (Anderberg, Lepp, Berglund, & Segesten, 2007).

According to Merleau-Ponty, we are our bodies and we experience the world through our bodies. When the body is changed, our access to the world is affected (Merleau-Ponty, 2011/1945). The result raises concern about how the care needs to adjust to older people's needs in terms of lived bodies. The encounter between the carer and the patient needs to be developed in order to get away from the view of the patient as an object. A broadened vision may open up for existential dimensions of what brings meaning to life. The statement from a woman who said that taking part in the TM made her “feel human”, indicates that participation in the TM is something more than mere presence. When the TM succeeds in creating feelings of being valued and treated with dignity in the patient, the outcome for the latter becomes something more than just “a meeting”, it goes beyond the pure TM situation, and enters into the caring and seeks its way into the lived body and the lifeworld.

One way of illustrating the intertwining between the lifeworld and the TM is through the patient's narratives, which involve descriptions of how they try to balance the facticity of life with belief in the future. According to Heidegger (1962), man is thrown into existence (Dasein), this means that she has to relate, to some extent, to facticities in life that she cannot fully control. Ageing and increasing vulnerability are facticities affecting life of the patients. Heidegger further elaborates on the concept of *mood*, meaning that a mood is always present in human existence. The mood is a part of our existence and as humans we are never free from mood. The patients in the study describe moods in relation to ageing and vulnerability. The patients describe that they do not always have explanations for the state of mood they were in, instead the mood as described by Heidegger “… assails us. It comes neither from the ‘outside’ nor from the ‘inside’, but arises out of Being-in-the-world, as a way of such Being” (Heidegger, 1962, p. 176).

Ageing and vulnerability are present in this facticity of life and when the mood is characterized by melancholy, the patients try to balance their lives by holding on to what Dahlberg, Todres, and Galvin (2009) describe as life projects. These offer a context and meaningful mission to fulfill. The family, a cat or engagement in social activities are examples of life projects described by the patients. Holding on to interests and having a mission to fulfill can offer rest in a difficult situation (Helvik, Cabral Iversen, Steiring, & Hallberg, 2011), create health and well-being (Dahlberg et al., 2009) and as a result indicates that maintaining life projects is one way of reducing vulnerability.

The facticity of life also involves the time into which the patients were, to use Heidegger's words, “thrown into existence”. The time and the society in which life is lived forms the present and age is not only the number of years you have lived, it is also about living one's life in a *generation* (Foss, 2011). The generation to which the informants “belong” has been in contact with health care services that are much more paternalistic and hierarchical than they are today. This can, according to Foss, affect the patients’ views on their possibilities of participating in their own care. Ekdahl et al. (2010) describe how the older patients perceived the hospital as “an institution of power” (p. 237), where they as patients were seen as less competent and therefore had to hand over decisions to the professionals. This study indicates that the patients’ possibilities of participating depend on the staff's attitude. Relational aspects are important for older patients in need of hospital care (Bridges, Flatley, & Meyer, 2010). The homelike feeling described by the patients in this study contributed to feelings of trust and security and encouraged them to take a place at the TM. However, the staff were sometimes described as uncomfortable and the patients’ presence appeared in these cases to have created insecurity among the staff. Instead of being supportive, the staff were described as silent and shy.

To be ill has a meaning to the person with the illness. Recognizing this and allowing the patient to talk about the meaning of illness and ageing can generate a better understanding for the patients’ situation (Svenaeus, 2011). Professionals need to be receptive to their patients’ own expertise in their lifeworld (Ranheim & Dahlberg, 2012) and it can be assumed that the patients need support to formulate concerns in relation to existential issues (Rydeman et al., 2012). Having the opportunity to tell one's life story appears to be essential for the patients in the study. Being invited to a TM in which the staff have the ability to confirm and listen to the patients’ narratives supports them in their struggle against vulnerability. Developing the round is much more than just changing its name to TM; it demands a change of perspective in which the focus on disease is changed towards a focus on the lived body, and as the results from this study indicate, it can be about providing space for existence in an institutional conversation.

## Methodological considerations

The interviews took place while the patients were still hospitalized; this means that considerations had to be taken concerning the fragile health of the patients. Engaging older people in research means that considerations must be given to the possibilities of physical impairment, frailty or loneliness (Pleschberger et al., 2011). The interviews had to be performed with adherence to the informants’ verbal and non-verbal signals of tiredness and exhaustion. Observations in combination with interviews were seemingly an appropriate method that created conditions for a more reflective and secure interview situation. The limited phenomenon also contributed to the richness of meanings in the interviews. The goal of phenomenological research is to describe a phenomenon via a search for essential meaning. The focus on the phenomenon can enable that the result, after careful consideration, can be transferred and applied to similar contexts.

The fact that the patients were in some kind of dependency, and that they often appeared to recognize the researcher as a member of the staff (even if the researcher introduced herself as researcher and wore private clothes) can have affected the informants’ eagerness to talk about things that might have discredited the care. The alternative to wait and conduct the interviews on a later occasion was not an alternative due to the fragile health of the patients.

## Conclusion and implications for practice

The patient has to surrender to care when vulnerability increases. Being a whole human with a unique life story in the encounter with care can provide vital strength and help in managing the challenge of ageing. Life as lived is present in the here and now situation, and it appears to be important to integrate the history in the current situation. Providing opportunities for life and existence in the institutional conversation, as for example in the TM, allows the patient to be seen in his/her uniqueness and can also support the patient in continuing life projects and contribute to a secure feeling when being discharged from hospital. In order to fully achieve this, a change from the patient as an object, to the patient as a lived body needs to take place. Creating space for the patient's life story as well as for his/her existence can lead health care development to a deeper dimension. This highlights the importance of a caring practice in which the older patient is offered the opportunity to become an active participant in the contexts where decision about the care is taken, as for example in the TM. The voice of the older patient needs to be given space, and in order to further develop a patient perspective, older patients need to be involved in the planning, implementation and evaluation of research and health care development.

## Conflicting interest and funding

The authors have not received any funding or benefits from industry or elsewhere to conduct this study.
